# From Intermittent Knee Pain to End-Stage Kidney Disease: An Atypical Presentation of Bilateral Kidney Dysplasia in an 11-Year-Old Girl

**DOI:** 10.1155/crpe/8856638

**Published:** 2025-09-01

**Authors:** Kristie Kim, Carlos Araya, Ryan Brogan

**Affiliations:** ^1^College of Medicine, University of Central Florida, Orlando, Florida, USA; ^2^Department of Pediatrics, Nemours Children's Hospital Florida, Orlando, Florida, USA

## Abstract

We describe the clinical presentation and evaluation of an 11-year-old girl with no reported past medical history, seen by her primary care physician for intermittent knee pain. Outpatient X-rays revealed findings concerning for rickets, prompting further evaluation with blood work. The patient was urgently referred to the emergency department due to abnormal laboratory results and was subsequently found to be in end-stage kidney disease with severe anemia, metabolic acidosis, and significant electrolyte abnormalities. Despite these abnormalities, she was hemodynamically stable with appropriate mentation, and her physical exam was only pertinent for left knee pain. Nephrology was consulted, and she was admitted to the pediatric intensive care unit (PICU) for electrolyte repletion and emergent hemodialysis. This case demonstrates an atypically benign presentation of end-stage kidney disease secondary to bilateral kidney dysplasia. It emphasizes the challenges of recognizing children with previously undetected congenital kidney disease and underscores the need for early detection in high-risk populations, including those affected by social determinants of health.

## 1. Introduction

Kidney dysplasia is a rare congenital abnormality and one of the major causes of chronic kidney disease (CKD) in children with a risk of progression to end-stage kidney disease (ESKD) over time [1]. Only 0.03%–0.16% of neonates on average are born with congenital anomalies of the kidney or urinary tract [2].

Kidney dysplasia is usually first detected via prenatal or postnatal ultrasound. In childhood, clinical symptoms associated with kidney dysplasia may include repeated urinary tract infections, urinary incontinence, flank or abdominal pain, edema, and elevated blood pressure [3]. However, elevated blood pressure is relatively infrequent in children with structural disorders such as renal dysplasia, whereas acquired (e.g., glomerular) renal diseases are associated with a higher prevalence of hypertension [4]. Prompt diagnosis of kidney dysplasia is important clinically, as delays in evaluation can result in increased morbidity due to untreated complications of CKD and a more rapid progression to ESKD. Unfortunately, clinical markers and symptoms of renal dysplasia are frequently subtle or absent. Definitive diagnosis of dysplasia may include a histologic analysis of the affected kidney; however, a biopsy or nephrectomy is rarely indicated or performed. Clinicians rely primarily on ultrasound imaging and specific characteristics such as the loss of corticomedullary differentiation, cortical thinning, and small kidneys as the sonographic equivalent of dysplasia [5].

In this case report, we describe a young patient with severe or advanced CKD secondary to bilateral kidney dysplasia, which remained unidentified until the patient presented to the general pediatrics office with a common pediatric chief complaint of knee pain.

## 2. Case Presentation

### 2.1. Patient Background

This 11-year-old patient was born full-term with no reported birthing complications or perinatal history, including prenatal imaging. The patient lives at home with her mother who is unemployed and experiencing financial difficulties. The mother states that the patient has appeared pale recently and has been wetting the bed. Otherwise, the patient has been healthy, attends fifth grade regularly with grades of As and Bs, and eats a varied diet including fruits, vegetables, proteins, and grains. Mother denies any family history of kidney disease or any prior concerning symptoms in the patient such as hematuria, urinary tract infections, or extremity edema.

### 2.2. Presentation, Diagnosis, and Outcome

The patient was seen by her primary care physician (PCP) for evaluation of bilateral knee pain that had been intermittent for several months. The pain was described as a deep ache that did not radiate and gradually worsened throughout the day. There were no other reported symptoms. An X-ray revealed findings concerning for rickets, prompting further evaluation with laboratory testing. The lab called the patient's PCP urgently due to significant abnormalities in the lab results, for which the patient was sent to the emergency room.

On arrival to the emergency room, the patient was found to be pale but hemodynamically stable and normotensive, with a normal blood pressure reading of 110/72 mmHg, pulse of 97 bpm, temperature of 98.4°F, respiratory rate of 24, and oxygen saturation of 100% on room air. Her physical exam was remarkable for failure to thrive. Her height and weight were both below the 1st percentile for her age, measuring 1.24 m and 21.5 kg, respectively. The rest of her physical exam was normal.

Her laboratory workup was consistent with uremia, severe anemia, anion gap metabolic acidosis, and severe hypocalcemia with secondary hyperparathyroidism of renal origin, all caused by advanced CKD (Table 1). The patient's estimated glomerular filtration rate (GFR) was calculated to be approximately 5 mL/min/1.73^2^ using the bedside Schwartz Pediatric formula placing her in the CKD Stage 5 category. An initial electrocardiogram (EKG) showed a normal sinus rhythm with a prolonged QT interval. A kidney ultrasound showed small echogenic kidneys measuring 4 cm, with poor corticomedullary differentiation consistent with kidney dysplasia. She was admitted to the pediatric ICU for correction of electrolytes and placement of a central venous catheter for initiation of acute hemodialysis.

The patient was treated with IV calcium gluconate, and then oral calcium carbonate and calcitriol to help control the profound hypocalcemia. She was also started on ferrous sulfate and erythropoietin (EPO) to treat the anemia. Although the patient did not present with symptomatic anemia, she received a transfusion of packed red blood cells due to the need for a dialysis catheter placement under anesthesia, which would otherwise be unsafe given her degree of anemia. She required sodium bicarbonate prior to dialysis to help improve the profound metabolic acidosis.

The diagnosis of severe CKD explained this child's presenting chief complaint of knee pain. It was secondary to high turnover bone renal osteodystrophy. The increased parathyroid concentration is the result of increased Fibroblast growth factor 23 concentration, phosphate retention, decreased calcium concentration, and reduced 1,25-dihydroxyvitamin D concentration.

After starting her treatments, the patient's mother reported that the patient was more energetic and had a better appetite. Her laboratory results showed significant improvement of severe anion gap metabolic acidosis, hypocalcemia, and resolution of hyperphosphatemia (Table 1). Severe uremia lingered for a few days until the hemodialysis clearance was progressively increased. The patient continued hemodialysis every other day during the hospitalization and was ultimately transitioned to an outpatient dialysis unit. Community resources including financial and emotional support were provided along with outpatient resources and close follow-up. The patient's hospital stay lasted a total of 10 days.

Since discharge, the patient reported feeling better, with just some fatigue, and tolerating dialysis without any events. She endorsed good appetite with resolution of her knee pains.

## 3. Discussion

Current literature emphasizes the need for early diagnosis and management of congenital kidney disorders to prevent complications of untreated CKD and more rapid progression to ESKD [5]. Delayed CKD diagnosis can lead to preventable morbidity and mortality and increased healthcare costs [6].

We present intermittent knee pain as the presenting manifestation of bilateral kidney dysplasia with ESKD in an 11-year-old girl. This case highlights the insidious presentation of severe CKD in an adolescent without any prior history of health issues, urinary symptoms, hypertension, or previously identified kidney anomalies.

Congenital anomalies of the kidney are the most common underlying diseases in pediatric CKD [7]. This case highlights the challenge of recognizing children with previously undetected congenital kidney disease. In a systematic review and meta-analysis of 19,339 children, Plumb et al. reported that late presentation occurred in 43% of the overall pediatric CKD population [8]. Plumb et al. also found that congenital anomalies, including renal dysplasia, had a 40% lower risk of late presentation compared to noncongenital causes.

Severe metabolic derangements of ESKD such as severe anemia, uremia, significant electrolyte abnormalities, severe metabolic acidosis, and secondary hyperparathyroidism with poor linear growth manifested in this case indicate that the patient's disease had progressed unnoticed for a long period of time. Pediatric CKD is characterized by markedly decreased quality of life and earlier death [6, 9, 10]. Known risk factors for mortality among children receiving kidney replacement therapy at a younger age include female sex, non-White race, and high estimated GFR at dialysis initiation [7]. However, it is difficult to adequately assess manifestations of pediatric CKD, such as renal osteodystrophy, resulting in very few high-quality findings to guide evidence-based practice [11].

This case underscores the pivotal role of the general pediatrician in the management of a child's comprehensive care. Even though knee pain in this age group may often be dismissed as “growing pains,” it was appropriately evaluated in this case, leading to earlier detection of concerning findings. Certain symptoms in children may suggest the need for referral to a nephrologist. Evidence of growth failure, such as height or weight velocity falling below the fifth percentile may indicate further evaluation by a nephrologist. Failure to thrive in a pediatric patient is an indication for laboratory evaluation. Persistent hypertension may also be a symptom; accordingly, it is important for the blood pressure to be checked at every pediatric visit. Additional red flags include persistent proteinuria or hematuria, unexplained electrolyte disturbances such as metabolic acidosis, and new-onset edema. A family history of kidney disease also warrants specialist evaluation. Early nephrology involvement helps to confirm CKD, optimize growth, and prevent long-term complications.

Given that the family in this case faced financial difficulties that may have affected their ability to attend routine visits with her pediatrician, this case highlights the critical role of a general pediatrician within the medical home and the need to address barriers to healthcare access and continuity. Socioeconomic factors, such as housing stability, food security, employment, and safe neighborhoods, have a big impact on how children with chronic conditions are diagnosed, treated, and supported over time [12]. Children from families with limited resources often face delays in diagnosis and challenges with treatment adherence, as well as lower rates of specialty-care utilization, particularly among those at or just above the poverty line [13]. Factors such as lack of transportation, inflexible work schedules, and difficulties navigating complex appointment systems disproportionately burden these families, further contributing to missed follow-up and fragmented care [14]. This case highlights how essential it is for pediatric providers to identify and help mitigate these barriers to improve outcomes for all children.

## Figures and Tables

**Table 1:** Laboratory test results of the case.

Laboratory test	On admission	At discharge	Reference
Comprehensive blood count			
Hemoglobin	5.3^↓^	8.0^↓^	11.3–13.4 g/dL
Hematocrit	16^↓↓^	24^↓^	32.3%–38.3%
Mean corpuscular volume	92^↑^	91^↑^	79.5–85.2 fL
Reticulocyte count	2.7	—	0.8%–2.8%
Comprehensive metabolic panel			
Sodium	138	140	134–143 mmol/L
Potassium	3.6	3.6	3.3–4.6 mmol/L
Chloride	109	105	96–109 mmol/L
TCO_2_	7^↓↓^	22	20–31 mmol/L
Blood urea nitrogen	117^↑^	39^↑^	7–17 mg/dL
Creatinine	11.7^↑↑^	4.8^↑↑^	0.31–0.61 mg/dL
Calcium	5.9^↓↓^	9.0	8.9–10.1 mg/dL
Alkaline phosphatase	1078^↑^	—	116–515 U/L
Blood gases, venous			
pH	7.13^↓↓^	7.34	7.34–7.43
pCO_2_	27^↓^	49	42–55 mmHg
pO_2_	155^↑^	50	30–50 mmHg
HCO_3_	10.4^↓↓^	26.4	24.0–28.0 mmol/L
Magnesium	1.5^↓^	2.0	1.6–2.2 mg/dL
Phosphate	6.1^↑^	3.2	2.5–6.0 mg/dL
Parathyroid hormone	3024^↑^	—	10.0–65.0 pg/mL
Erythrocyte sedimentation rate	71^↑^	—	0–20 mm/hr

^↓^Low value.

^↓↓^Critically low.

^↑^High value.

^↑↑^Critically high.

## Data Availability

The data that support the findings of this study are available on request from the corresponding author. The data are not publicly available due to privacy or ethical restrictions.
